# Are There Bad ICU Rooms? Temporal Relationship between Patient and ICU Room Microbiome, and Influence on Vancomycin-Resistant *Enterococcus* Colonization

**DOI:** 10.1128/msphere.01007-21

**Published:** 2022-02-02

**Authors:** Daniel E. Freedberg, Miles Richardson, Mary Nattakom, Jacky Cheung, Elissa Lynch, Philip Zachariah, Harris H. Wang

**Affiliations:** a Division of Digestive and Liver Diseases, Columbia University, New York, New York, USA; b Department of Medicine, Columbia University, New York, New York, USA; c Department of Systems Biology, Columbia University, New York, New York, USA; d Department of Pediatrics, Columbia University, New York, New York, USA; e Department of Pathology and Cell Biology, Columbia University, New York, New York, USA; f Integrated Program in Cellular, Molecular, and Biomedical Studies, Columbia University, New York, New York, USA; University of Michigan-Ann Arbor

**Keywords:** gut microbiome, intensive care unit, vancomycin-resistant *Enterococcus*, healthcare-associated infections

## Abstract

The gut microbiome of an individual can shape the local environmental surface microbiome. We sought to determine how the intensive care unit (ICU) patient gut microbiome shapes the ICU room surface microbiome, focusing on vancomycin-resistant *Enterococcus* (VRE), a common ICU pathogen. This was an ICU-based prospective cohort study. Rectal swabs were performed in adult ICU patients immediately at the time of ICU admission and environmental surface swabs were performed at five predetermined time points. All swabs underwent 16S rRNA gene sequencing and culture for VRE. 304 ICU patients and 24 ICU rooms were sampled (5 longitudinal samples per ICU room). Spatially adjacent ICU rooms were no more microbially similar than nonadjacent rooms. Microbial signatures within rooms diverged rapidly over time: in 14 days, ICU rooms were as similar to other ICU rooms as they were to their prior selves. This divergence over time was more pronounced in rooms with higher patient turnover. Examining VRE status by culture, patient VRE gut colonization had modest agreement with room surface VRE (kappa statistic 0.36). There were no ICU rooms that consistently cultured positive for VRE, including those that housed VRE positive patients. Individual ICU patients had a limited impact on ICU room surface microbiome, and rooms diverged similarly over time regardless of patients. Patient VRE gut colonization may have a modest influence on room surface VRE but there were no “bad rooms” that consistently cultured positive for VRE. These results may be useful in planning infection control measures.

**IMPORTANCE** This study found that intensive care unit (ICU) room microbial signatures diverged from their baseline quickly: within 2 weeks, individual ICU rooms had lost distinguishing characteristics and were as similar to other ICU rooms as they were to their former selves. Patient turnover within rooms accelerated this drift. Patient gut colonization with vancomycin-resistant *Enterococcus* (VRE) was associated with ICU room surface contamination with VRE; again, within 2 weeks, this association was substantially diminished. These results provide dynamic information regarding how patients control the microbiota on local hospital room surfaces and may facilitate decision making for infection prevention and control measures targeting VRE or other organisms.

## INTRODUCTION

The gut microbiome of hospital patients shapes the local environmental microbiome, and this is important in the intensive care unit (ICU), where health care-associated infections are a leading cause of death (1–3). In the ICU, environmental surface colonization with pathogenic bacteria is common, despite decontamination efforts. ICU surface colonization is nontrivial, because patients housed in an environment containing pathogens are more likely to become colonized (4). Once the gut is colonized with pathogenic bacteria such as vancomycin resistant *Enterococcus* (VRE), patients are more likely to become infected with the same organism and to die (5).

In newly opened hospitals or hospital units, the initial room surface is nonsterile but relatively free from pathogens such as VRE (6). Introduction of patients rapidly changes the environmental surface microbiome so that it more closely resembles the patient microbiome (2, 6). How the environmental microbiome changes over time in established hospital ICUs, and how this depends on patients and their gut colonization, is not certain.

VRE causes 18,000 health care-associated infections annually in the United States., second only to E. coli (7, 8). VRE is present in the gut in one third of medical ICU patients at the time of ICU admission (9), and another 20% of ICU patients may acquire VRE gut colonization during long-term ICU stays (10). When patients enter an environment that is already VRE colonized, they are at increased risk for becoming VRE colonized (4, 11, 12). While these and other studies have shown how environmental contamination with VRE contributes to patient colonization, the reverse question—how do patients spread VRE into the local environment?—remains incompletely understood.

This study described the environmental surface microbiome of the medical ICU and assessed how it was influenced by patients over time. By combining patient samples taken immediately at the time of ICU admission with longitudinal ICU room samples taken at intervals over 4 months, it sought to understand the extent to which patients might alter the trajectory of their local room surface microbiome. Separately, we focused on a specific organism, VRE. VRE was cultured in patients and on ICU room surfaces to test how patient VRE colonization status might influence ICU room VRE status over time.

## RESULTS

### Patient population.

**FIG 1:**
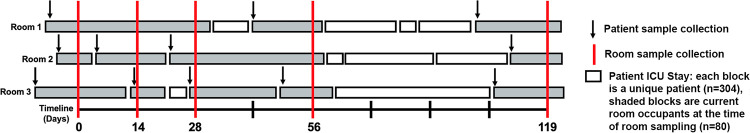
Schematic representation of the timing of room and patient sample collections during the study period. Rooms were sampled at five time points with approximate doubling of the time interval (day 0 and then on Days 14, 28, 56, and 119). Patients were sampled immediately at the time of ICU admission, when they were transferred from their transportation stretcher into their ICU room bed. On the schematic, each rectangle represents a unique patient, and each row represents a unique ICU room. Shaded rectangles represent patients who were in the room at the time of room sampling. A total of 24 ICU rooms were sampled at five time points (120 room environmental samples) and 304 ICU patients, although only 80 of these patients were in the room at the time of room sampling.

Twenty-four unique ICU rooms were sampled simultaneously at 5 time points (120 environmental samples) while they were occupied by patients. A total of 304 ICU patients were sampled, with all patients sampled immediately at the time of ICU admission (i.e., patient sampling always prior to room sampling). Because some patients remained in the same rooms during multiple rounds of room sampling (see Fig. 1), there were 80 unique patients who were in the rooms at the time when the room samples were collected. Analyses focused on these 80 patients based on the premise that patients who occupied the rooms most recently would be most influential on the room surface microbiome. Among these 80 patients, median age was 59 years old (interquartile range [IQR], 48 to 70). Almost 80% of patients were admitted for sepsis and/or respiratory failure and 83% received antibiotics within the 24 h preceding ICU admission (Table S1).

10.1128/msphere.01007-21.2 TABLE S1 Patient characteristics for 80 patients occupying the ICU rooms at the time when the rooms were sampled. Download Table S1, PDF file, 0.1 MB. Copyright © 2022 Freedberg et al. 2022 Freedberg et al. https://creativecommons.org/licenses/by/4.0/ This content is distributed under the terms of the Creative Commons Attribution 4.0 International license.

### Microbial patterns.

**FIG 2:**
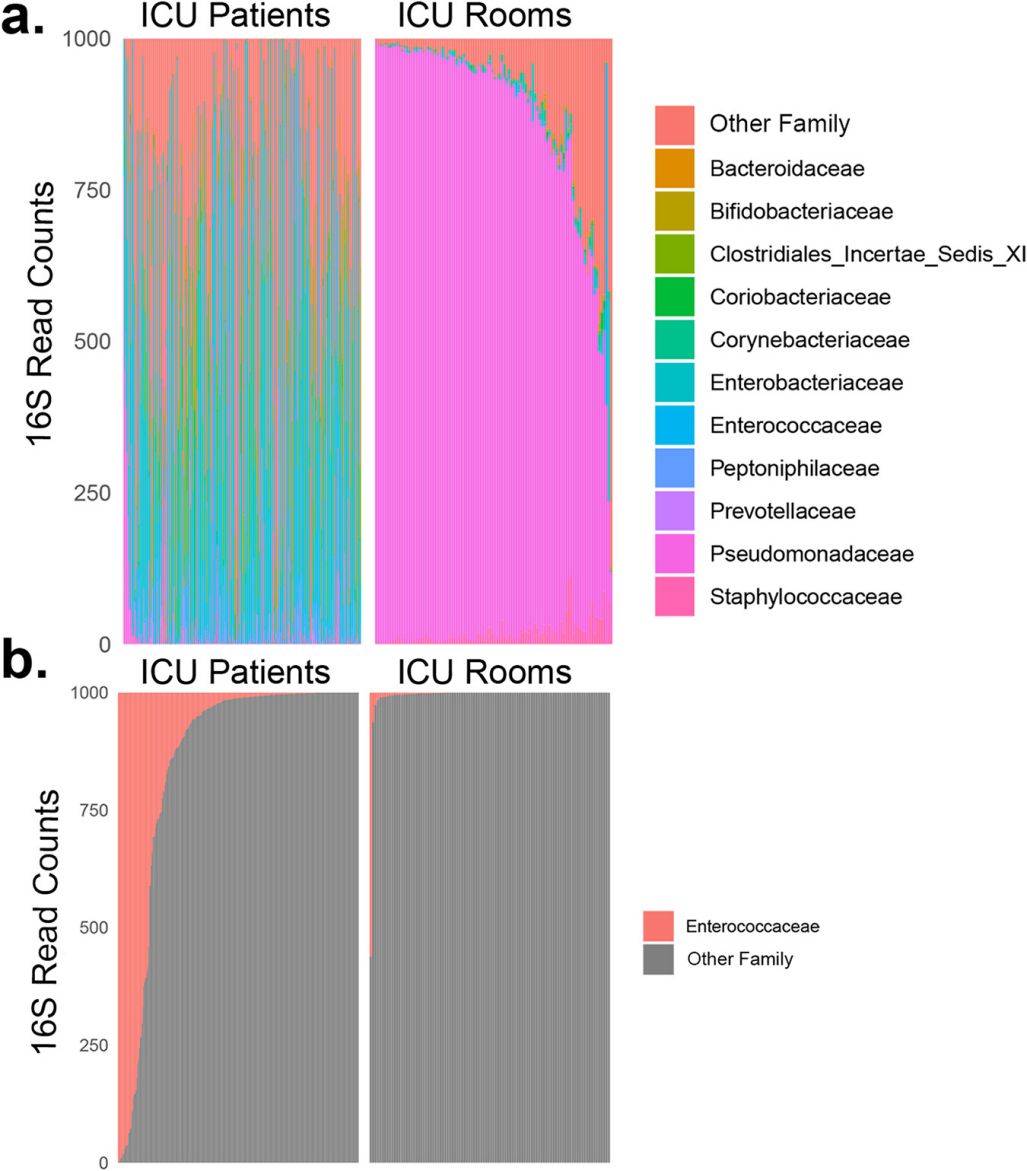
Microbial community patterns in ICU patients and ICU rooms. Relative abundance is shown for the most common taxa at the family level (a) and highlighting *Enterococcaceae* only (b). Patients are on the left and ICU rooms are on the right. The top panels are organized from left to right by decreasing relative abundance of *Pseudomonaceae* and the bottom panels are organized by decreasing relative abundance of *Enterococcaceae*. Comparing rooms versus patients, the rooms were overrepresented in *Pseudomonaceae* and the patients were overrepresented in *Enterococcaceae*.

*Microbial Community Patterns in Patients and ICU Rooms:* First, we profiled ICU patients and rooms to assess broad differences. The microbiome of ICU room environmental surfaces differed radically from patients’ gut microbiome, making comparisons of specific taxa difficult. ICU room surfaces were dominated by *Pseudomonaceae* (median relative abundance [RA] 93%, IQR 82% to 96%) compared to patients (median RA 0%, IQR 0% to 0%, Mann-Whitney U test *P* < 0.01 for rooms versus patients, Fig. 2a). Conversely, *Enterococcaceae* were overrepresented in patients compared to rooms (median RA 1.2%, IQR 0.2% to 12% for patients versus 0%, IQR 0% to 2% for rooms, *P* < 0.01, Fig. 2b). Efforts to identify specific patient-to-room microbial signatures using SourceTracker and other tools failed, in part because the patients and rooms were so dissimilar.

*Microbial Similarity of Neighbor ICU Rooms:* To assess dispersion of the microbiome within the ICU as a whole, we tested the microbial similarity between neighbor ICU rooms (ones that shared a common wall) and examined how this changed over time. Pairwise weighted unifrac distance was computed between neighbor rooms, and this was compared against the weighted unifrac distances between nonneighbor rooms; the neighbor and nonrooms were similar at baseline (*P* = 0.29).

### Temporal relationships.

**FIG 3:**
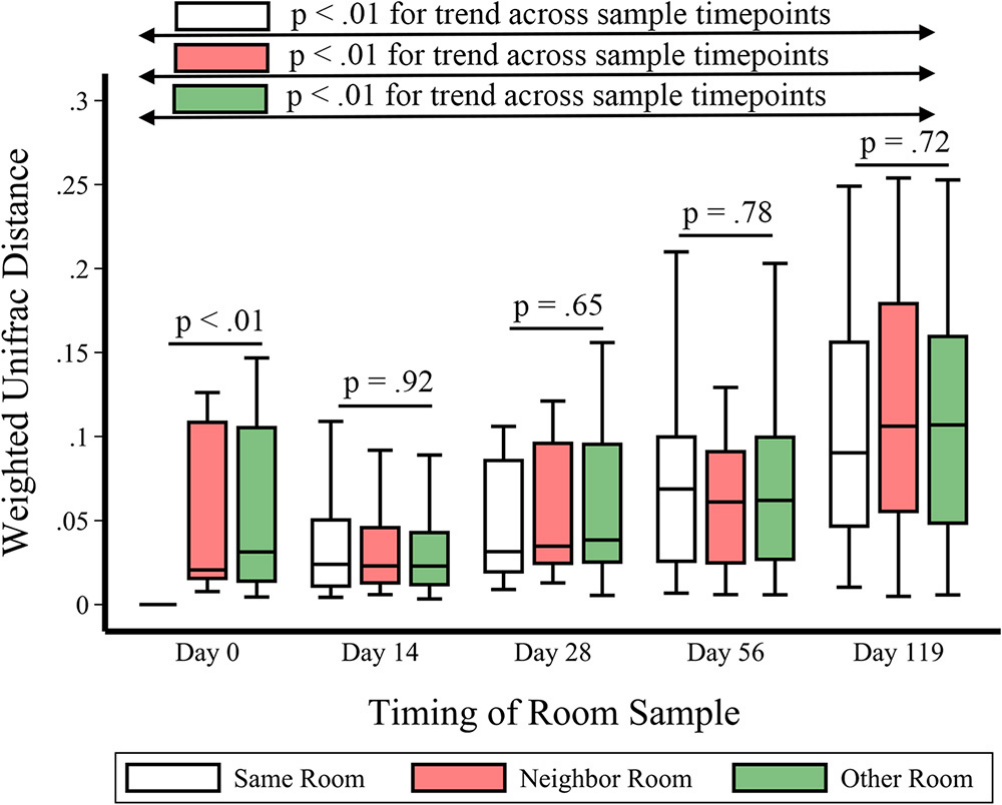
Weighted unifrac distances over time are shown within the same rooms (white), within neighbor rooms (red), and within nonsame nonneighbor rooms (“other rooms,” green). At any given time point, there were no significant differences comparing self versus neighbor versus other rooms, using the baseline/Day 0 sample as the reference point. However, over time, there were increasing weighted unifrac distances for all three comparison types (Jonckheere-Terpstra trend *P* < 0.01).

*Similarity Over Time:* Next, we assessed changes in rooms over time. Pairwise within-room weighted unifrac differences were computed based on sampling time interval (e.g., Room A at baseline with Room A at day 14, Room A at baseline with Room A at day 28, etc.). Similarly, pairwise weighted unifrac distances were computed for rooms with neighbor rooms and for rooms with nonself, nonneighbor rooms over time (e.g., Room A at baseline with Room B at baseline, Room A at baseline with Room B at day 14, etc.). For all of these comparisons, the baseline room sample was used as the reference point. A drift was observed toward divergence (i.e., increasing weighted unifrac distance) over time (Jonckheere-Terpstra trend *P* < 0.01, Fig. 3). The rate of divergence was similar when comparing rooms to their former selves, rooms to their neighbors, or rooms to nonneighbors (Fig. 3).

**FIG 4:**
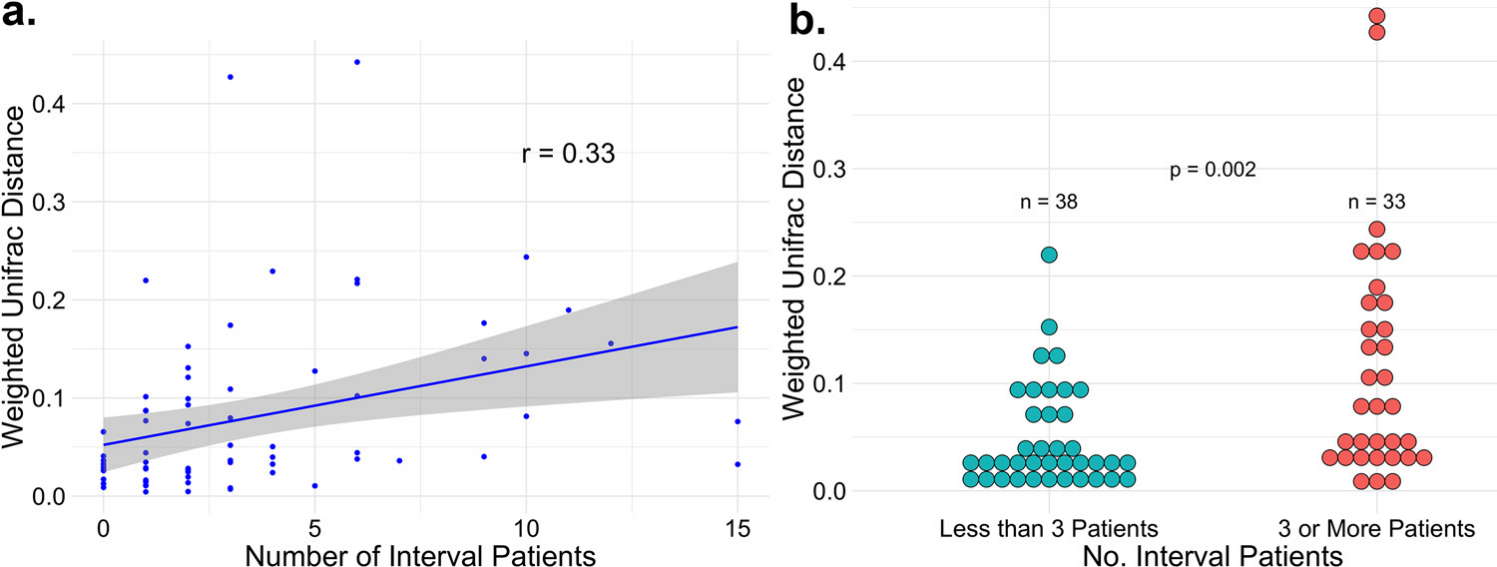
Patient turnover and room changes over time. (a) Within-room weighted unifrac distance was computed for all rooms and correlated with the number of unique patients in the room in between samples. (b) As an alternative way of visualizing these data, rooms were classified as high turnover (≥3 interval patients between samples) versus low turnover (<3 interval patients between samples) and within-room weighted unifrac distance from day 0 to day 14 was compared. After 14 days, macro-environmental factors (e.g., seasonal changes) may be of increasing importance and may overwhelm local environmental factors.

*Impact of Patient Turnover on Within-Room Changes over Time:* We hypothesized that higher patient turnover (i.e., more patients in the room between room samples) would associate with more rapid divergence in the room environment over time. To test this, we correlated patient turnover with the within-room weighted unifrac distances between sample time points. There was a modest but statistically significant association (Figure 4a, Spearman *r* = 0.33, *P* < 0.01). As an alternative way of visualizing the data, rooms were divided into high patient turnover rooms (≥3 unique patients between day 0 and day 14) and low patient turnover rooms (<3 unique patients). The cutoff three patients was selected because it was the median number of unique interval patients. Using this classification, the high turnover rooms had significantly greater divergence compared to the low turnover rooms (Figure 4b, Mann-Whitney *P* < 0.01).

### Vancomycin resistant *Enterococcus* (VRE).

*VRE Colonization Status of Patients and Rooms:* We focused on the patient-room interaction with respect to a single organism, VRE. Using selective culture, VRE was classified as present or absent in both rooms and patients. Overall, 35% of room environmental samples and 28% of patient samples grew VRE. No rooms were VRE culture positive at every time point and there was a modest decrease in room VRE colonization over time (p for trend <0.01). There were 80 unique patients who occupied the rooms at the time of room sampling, with the rooms sampled at median interval of 4.5 days (IQR 2.0 to 10.1) after the patients. When patients were VRE colonized, 14/22 (64%) room surfaces grew VRE whereas when patients were not VRE colonized 14/58 (24%) rooms grew VRE (Table 1, Cohen’s kappa 0.36). The converse of this was that when patients were VRE negative, 44/58 (76%) of room surfaces did not grow VRE whereas when patients were VRE positive, 8/22 (36%) of room surfaces did not grow VRE (Table 1). When VRE colonies were counted from the composite room swabs, there was no association between room VRE abundance and patient VRE positivity.

**TABLE 1:** Association between patient vancomycin-resistant *Enterococcus* (VRE) gut colonization and corresponding room environmental surface VRE status

		Room VRE status		Proportion of Rooms (+)	Proportion of Rooms (−)
		Positive (+)	Negative (−)		
Patient VRE Status	Positive (+)	14	8	64%	36%
	Negative (−)	14	44	24%	76%

Cohen’s kappa statistic for patient-room agreement = 0.36.

**FIG 5:**
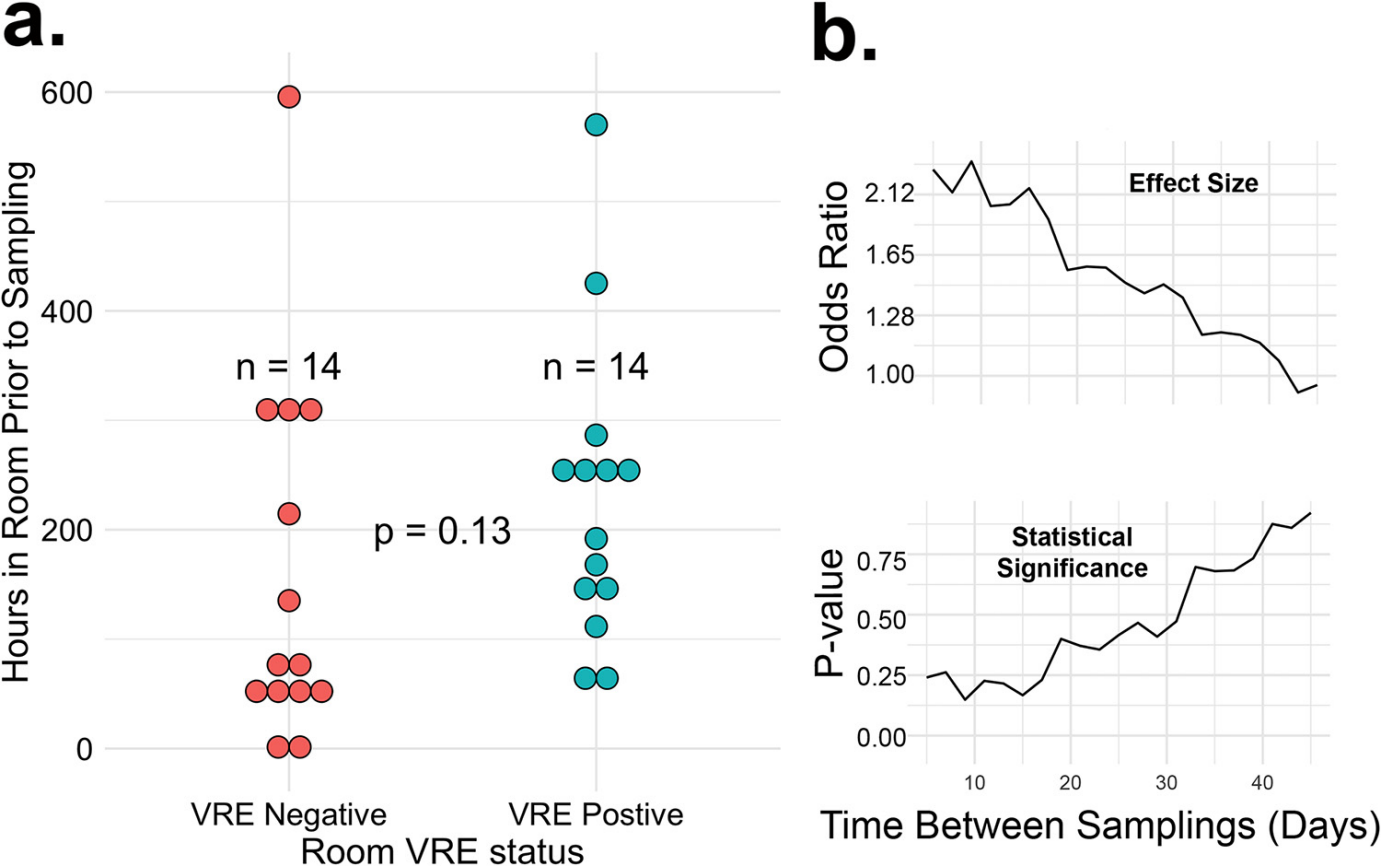
(a) VRE status of patients and rooms. The data shown is for 28 VRE positive patients currently in the room when the room was sampled. The number of hours elapsed between sampling of VRE colonized patients and sampling of ICU rooms is shown in relation to room VRE colonization status. (b) Predictive value of patient VRE status for room surface VRE status. These data are for all 304 patients, regardless of whether they occupied the room at the time when the room surfaces were sampled. The room VRE status was modeled as the outcome with patient VRE gut colonization status and the time between patient and room sampling as predictor variables. The upper and lower panels show the odds ratio and *P*-value on the *y* axes, respectively, as a function of the time between when the patient was sampled and when the room was sampled (X-axis). The analysis was terminated at 40 days, after which there was minimal confidence in the estimates.

*Dependence of Room VRE Colonization on Sampling Time Interval:* Last, because the rooms were sampled at different intervals after the patients began occupying them, we sought to determine whether duration of room occupancy influenced the patient-room VRE relationship (i.e., if a VRE positive patient occupied the room for a longer period of time, were the room surfaces more likely to grow VRE?). First, we examined the duration of room occupancy among 28 VRE positive patients who occupied the room at the time of room sampling, and tested whether room occupancy duration differed based on room VRE status. Patient occupancy of the room was longer when the room was VRE positive, concordant with the patient (median 9 days, IQR 6 to 11) compared to when the room was VRE negative and was discordant with the patient (median 3 days, IQR 2 to 12) but this difference was not statistically significant (Figure 5a and *P* = 0.13).

To understand if the effect of patient VRE status on room VRE status was time dependent, we developed a generalized linear mixture model. In this model, VRE positivity of the room was the dependent variable, with patient VRE status and the time difference between sampling each surface as fixed effects, and room as a random effect. This model incorporated data from all patients who occupied the rooms and not only the current room occupants at the time of room sampling. We then subsampled the data at various intervals: first all samples collected within 5 days of each other, then 7, etc. up to 45 days. We trained the model on each of these subsets. The results are shown in Fig. 5b in this model, patient VRE positivity was associated with an odds ratio (OR) of 2.35 (95% CI 0.56 to 9.74) for VRE room positivity when the time between patient and room samples was 5 days, with the strength of the association decreasing to OR 1.57 (95% CI 0.58 to 4.22) when the time between samples was 20 days, and OR 1.06 (95% CI 0.48 to 2.02) when the time between samples was 40 days.

## DISCUSSION

This prospective study performed in single-occupancy medical ICU rooms found that the ICU room surface microbiome slowly diverged from its baseline over 120 days. Rooms had little individuality with respect to this divergence: within 14 days, the surface microbial community of a given ICU room was as similar or dissimilar to itself 14 days prior as it was to the surface microbial community of a neighbor ICU room, or to the surface microbial community of a nonneighbor, nonself room. This interesting null finding suggests that ICU room surfaces experience a high degree of turnover, which serves to make the microbial community almost unrecognizable in a relatively short period of time. This view of the ICU as a shared yet rapidly changing ecology is similar to prior studies characterizing the hospital micro-environment (2) and differs somewhat from the view that individuals disperse a highly personalized microbial cloud (13). Supporting this vision of the ICU is the finding that there was an overall decrease in ICU VRE positivity over time.

Multiple factors could contribute to drift in the microbial community of ICU room surfaces over time. Seasonal shifts may alter solar exposure, temperature, and airflow through centralized heating or cooling systems that are shared across rooms within the same ICU. In this stdy, use of composite room surface swabs may have obscured stability in microbial composition within niche micro-environments such as sink drains (14). Patients are another factor because each newly admitted ICU patient brings a unique set of microbes that are then dispersed into the room through patient secretions, including stool. At the same time, rooms were thoroughly cleaned between patient admissions (referred to as a “terminal cleaning”), so each new admission was preceded by an attempt to purge the room from pathogens. The ICU room surface microbial community became more dissimilar as the number of patients and terminal cleanings increased between room sampling time points. This shows that patient turnover can lead to increased room community dissimilarity. However, because patient turnover and terminal cleanings are inseparable in this data set, we cannot determine which of these factors is more important. In prior studies, thorough environmental cleaning reduces the risk for patient colonization or infection with VRE, and it seems likely that the same disruption serves to shift the room microbiome over time (15–17).

Does the same decay in microbial community composition also apply to VRE? When VRE positive patients are prior room occupants, subsequent patients admitted to the same rooms may face increased risk of acquiring VRE (18). The current room occupant’s VRE status very modestly agreed with the room’s VRE status in this study (Cohen’s kappa 0.36). Further, there was some evidence that this agreement may depend on time. When the VRE status of the current room occupant was discordant with the VRE status of the room, the median time of patient room occupancy was 3 days versus 9 days when the current room occupant and the room were both VRE positive. When looking across all patients (not just the current room occupant), there were no associations between patient and room VRE status. Like our finding related to room turnover, this again supports the conclusion that terminal cleanings performed after patient discharge do have some effect on room colonization.

This study has strengths. It involved samples drawn from the patient as well as from the local hospital room surface micro-environment, thereby permitting us to link patient-level data to specific room surface data. It investigated a microbe (VRE) of established clinical significance. And it used culture for VRE as well as sequencing data, providing reassurance that viable organisms were being studied. The study also has limitations. It was performed at a single center, and all ICUs are likely to have unique characteristics in terms of local climate, cleaning practices, patient demographics, and VRE rates. It was not designed to examine non-VRE pathogens such as multidrug-resistant Gram negatives, although these organisms are also of clinical significance. Room cleanings were not systematically documented, and therefore the impact/durability of room cleaning could not be directly studied. Although rooms were sampled longitudinally, patients were sampled at a single time point and we are therefore unable to comment on whether patient gut microbiome shifts are reflected by corresponding shifts in the ICU room surface microbiome composition. Last, patient and room samples were not collected simultaneously. This is an important limitation that precludes a complete assessment of the patient-room dynamics in VRE transmission. In an ongoing study (NCT03865706) patients and their ICU rooms are sampled simultaneously.

### Conclusions.

In sum, this prospective medical ICU cohort study found that the overall microbial community profile of single-occupancy ICU room surfaces drifted away from its baseline without significant room individuality. The gut microbiota of the patient occupying the room did influence the room microbiota, but this effect dwindled to nothing over 14 days. Patient turnover—and the room terminal cleanings that went along with this—was the factor most associated with microbial changes within ICU room surfaces over time. Current infection prevention and control efforts often focus on terminal cleanings under the rationale that each individual room must be made clean for the benefit of the incoming patient. Our results suggest an alternative approach that focuses on the pathogen burden of the ICU as a whole. The rooms likeliest to be colonized with VRE are those occupied within 14 days by a patient who is colonized with VRE. Cleaning such rooms early—while still occupied by a VRE positive patient—may have more positive impact on the overall ICU than terminally cleaning them, after the VRE positive patient has had the opportunity to disperse VRE into the ICU. The logistical challenges of adequately cleaning rooms while occupied by patients are nontrivial and need to be balanced against potential benefits. Nonetheless, future infection prevention and control efforts may wish to test strategies that focus on targeted interval rather than nonspecific terminal room cleanings. In the short term, our results provide reassurance that there are unlikely to be “bad rooms” which are consistently enriched with VRE.

## MATERIALS AND METHODS

### Study design and sampling.

This study was conducted from April 1, 2017 to September 30, 2017. All of the ICU rooms within the participating ICUs were sampled simultaneously at five time points over 4 months (Fig. 1). Patients were all sampled at the time of ICU admission, immediately after they were transferred from their transportation bed into their ICU bed. The Institutional Review Board of the Columbia University Irving Medical Center approved the study (IRB number AAAN7352).

### Patient swabs.

Patients ≥18 years old who were admitted to either of two medical ICUs were eligible for the study if a VRE rectal swab had been captured for them. Patient clinical data were retrieved from the electronic medical record for descriptive purposes. The study used leftover (waste) VRE surveillance swabs and was performed at a time when VRE surveillance swabbing was done routinely on all medical ICU patients. Flocked nylon swabs (Copan Diagnostics) were performed by patients’ nurses, who were instructed to rotate the swab in the rectum and to use fecal soilage to verify that swabbing was adequate.

### ICU room swabs.

ICU room sample intervals were designed such that there was an approximate doubling in each time interval between samples: baseline/study day 0 and subsequently on day 14, 28, 56, and 119. Because ICU room surfaces can have a low bacterial load, a large moistened sponge-type swab (3 M number SSL10NB) was used to collect composite bacteria from several ICU surfaces and individual surfaces were not analyzed. To select which surfaces were swabbed, we referenced prior studies which have determined which ICU surfaces are most frequently touched by staff (19). These surfaces were sampled consecutively with the composite swab, spiraling out from the patient as previously described (6). Each surface was brushed for 60 s in the following order: (1) bed rails, (2) infusion pumps/television remote/nursing call button, (3) ICU room sink lip, and (4) floor directly beneath the patient’s bed, at the level of the head. Swabs were then milked to produce 1–2 mL of fluid for VRE culture and sequencing.

### ICU room cleaning protocol.

All 24 ICU rooms in the study were single-occupancy and each room contained its own sink and commode. Routine chlorhexidine bathing was not performed during the study period. A room terminal cleaning was performed by environmental services (EVS) after patient discharge in all the rooms using wipes containing 0.63% sodium hypochlorite, equivalent to a 1:10 bleach dilution. This terminal cleaning protocol specified surface disinfection for a minimum 4 min of wet surface time. Cleaning performed while rooms were occupied by patients was with wipes containing 0.5% quaternary ammonium for a minimum 2 min of wet surface time. No institutional changes were implemented in cleaning protocol during the course of the study. The room surfaces targeted for cleaning and the frequency of routine cleaning is further described in the Supplemental Table S2.

10.1128/msphere.01007-21.3 TABLE S2 The surface cleaning protocols and frequencies that were in place for the duration of the study. Rooms were cleaned after patient discharge using wipes containing 0.63% sodium hypochlorite, equivalent to a 1:10 bleach dilution, for 4 minutes of wet surface time. Cleaning performed while rooms were occupied by patients was with wipes containing 0.5% quaternary ammonium for 2 minutes of wet surface time. All of the 24 ICU rooms sampled were single-occupancy, and no routine chlorhexidine bathing was performed during the study period. No institutional changes were implemented in cleaning protocol during the course of the study. Download Table S2, PDF file, 0.7 MB. Copyright © 2022 Freedberg et al. 2022 Freedberg et al. https://creativecommons.org/licenses/by/4.0/ This content is distributed under the terms of the Creative Commons Attribution 4.0 International license.

### VRE culture.

Patient and room swabs were collected, placed directly into Amies medium, and immediately carried to the laboratory where they were streaked onto plates of chromogenic media impregnated with 6 μg/mL of vancomycin (Remel) without any cold storage period. Plates were incubated at 37°C under aerobic conditions for 24 h and classified as positive if three or more colonies showing appropriate color and morphology were present on each selective plate.

### 16S rRNA gene sequencing.

Samples used for sequencing were collected as described above and immediately flash frozen at minus 80°C for 16S sequencing at the end of the study. Batched DNA extraction was performed using the PowerFecal DNA isolation kit (Mo Bio, Carlsbad, CA). PCR was performed targeting the V4 hypervariable region of the 16S rRNA gene with primers derived from the human microbiome project (20). Samples were pooled and purified with the QIAquick PCR kit (Qiagen, Valencia, CA) and library quantification performed using a KAPA Library Quantification kit (Kapa Biosystems, Wilmington, MA). Sequencing of the 16S rRNA gene V4 region was then performed using the Illumina MiSeq platform (Illumina, San Diego, CA). Using the DADA2 (21) pipeline, reads were filtered with no ambiguous bases, max expected error of 2, and were truncated to 150 bp. Reads were then processed and bimeras were removed. Taxonomy was assigned using the RDP classifier and the RDP training set version 16 (22). Chloroplast and mitochondrial DNA were removed, and each sample was rarefied to 1000 reads. The phylogenetic tree used for Unifrac analyses was generated using Phangorn, initialized with a neighbor joining tree, and updated using a Generalized Time Reversal (GTR) maximum likelihood tree (23).

### Measuring changes in icu rooms over time.

Weighted unifrac distance was selected to assess change in ICU rooms over time because weighted unifrac is a highly established measure of β-diversity and interfaces well with the analytic pipeline that we used (24). Weighted unifrac distance has been demonstrated to perform well at comparing environmental microbiome community data over time (24).

### Statistical approach.

Statistical testing was performed using STATA version 16 or R. Plots were made using ggplot2 (25). When continuous data were compared, it was first assessed for normality so that appropriate tests could be performed (e.g., *t* test or Mann-Whitney U-test test as appropriate). To assess whether patient VRE colonization status predicts ICU room VRE status, Cohen’s kappa statistic (26) for agreement was selected because (unlike, e.g., McNemar’s test) (27) it recognizes sample size and makes fewer assumptions regarding the independence of paired data. To examine how patient time spent in the room might affect the room VRE status, a generalized linear mixture (GLM) model was used. This GLM model had ICU room VRE status as the outcome variable and patient VRE status and the time interval from patient sampling to room sampling as predictor variables, modeled as fixed effects and the room modeled as a random effect. These and all other statistical tests were conducted two-sided at the α = 0.05 level of significance.

### Data availability.

Sequencing data and sample information are available from Qiita accession number 13832 and EBI accession number ERP130386.
