# Supplementary material for: Are There Bad ICU Rooms? Temporal Relationship between Patient and ICU Room Microbiome, and Influence on Vancomycin-Resistant Enterococcus Colonization
Source: mSphere. 2022 Feb 2;7(1):e01007-21. doi: 10.1128/msphere.01007-21 (PMC8809377; doi:10.1128/msphere.01007-21)
Supplement: TABLE S1 [file msphere.01007-21-st001.pdf]

Supplemental Table 1. Patient characteristics for 80 patients occupying the ICU rooms at the time when the rooms were sampled.

**Characteristics**	**N**	**Proportion**
Age (years)		
< 40	16	20%
40 to 59	25	31%
60 to 69	17	21%
≥ 70	22	28%
Sex		
Male	38	48%
Female	42	53%
Duration of hospitalization prior to ICU admission		
≥ 28 days	13	16%
1 to 27 days	46	58%
Admitted to ICU from ER	21	26%
Reason for ICU admission		
Sepsis and/or respiratory failure	63	78%
Coma/neurologic	5	6%
Hypovolemic shock	3	4%
Arrhythmia	2	3%
Other	7	9%
Receipt of antibiotics within 24 hours before ICU admission		
None	10	13%
Narrow spectrum only	4	5%
Broad spectrum with or without narrow spectrum	66	83%
Treatments received within 24 hours after ICU admission		
Broad-spectrum antibiotics	73	91%
Mechanical ventilation	40	50%
Vasopressors	40	50%
Proton pump inhibitors	30	38%
Hemodialysis	14	18%
ECMO	5	6%
SOFA score at ICU admission		
0 to 6	38	48%
7 to 9	25	31%
≥ 10	17	21%

ECMO: extracorporeal membrane oxygenation; ER: emergency room; ICU: intensive care unit; SOFA: sequential organ failure assessment.
