# Supplementary material for: Are There Bad ICU Rooms? Temporal Relationship between Patient and ICU Room Microbiome, and Influence on Vancomycin-Resistant Enterococcus Colonization
Source: mSphere. 2022 Feb 2;7(1):e01007-21. doi: 10.1128/msphere.01007-21 (PMC8809377; doi:10.1128/msphere.01007-21)
Supplement: TABLE S2 [file msphere.01007-21-st002.pdf]

NYPH CSPD Cleaning Standards and Responsibilities

Surface/Item	Frequency	Dept. Responsible	Recommended Product	Details
**Decontamin ıtion*/ Ante Room (site specific)** *must have dedi :ated cleaning cart and supplies for this area*				
Floors, Sinks				
**Sweep floors and baseboards**	Once a day	EVS	Microfiber mop	
**Wet mop floors**	Once a day	EVS	Microfiber mop and Hospital approved disinfectant	
**Auto-Scrub Floors**	Twice a week	EVS	Hospital approved disinfectant	Move all furniture from side to side. Once finished, Power Washer is stored properly
**Wash Anti-slip mats**	Once a day	CSPD	Put through cart washer	
**Wash Anti-fatigue mats**	Once a day	CSPD	Put through cart washer	
**Disinfect all sinks, faucets and backsplashes**	Once a day	EVS	Bleach Wipes	
**Wipe Soap and Purell Dispensers**	Once a week	EVS	Hospital approved disinfectant	Fill as needed
**Maintain internal Floor and Sink drains**	As needed	EVS / Facilities	Hospital approved disinfectant	EVS to notify Facilities with Work Order
**Mop under Ultrasonic Washer**	Once a month	*EVS Supervisor and *CSPD Supervisor	Hospital approved disinfectant	*Coordinated between supervisors **DO NOT MOVE WITHOUT CSPD**
Ceiling, Walls, Doors				
**Wash walls**	Once a day	EVS	Hospital approved disinfectant	Use wall washing tool; Start at top and work down
**Wash doors, door panel, automatic door panel, door handles, and glass**	Once a day	EVS	Bleach and Glass Cleaner	Glass to be cleaned with glass cleaner, door with bleach.
**Clean and/or Replace Ceiling Tiles**	Once a month	EVS or Facilities (see detail)	Hospital approved disinfectant	EVS to clean; Facilities to replace tiles when wet within 72 hours. Use Hepa Cart
**Dusting external Light Fixtures and/or covers**	Once a day	EVS	High Duster	
**Wipe Air vents (internal)**	As needed	EVS / Facilities		EVS to notify Facilities with Work Order
**Dust Air Ducts, Vents (external covers)**	Once a month (more often if visibly soiled)	EVS	High Duster	
Shelving, Workstations				
**Wipe Cabinets (exterior)**	Once a day	EVS	Hospital approved disinfectant	
**Wipe Counters**	Once a day	EVS	Hospital approved disinfectant	
**Wipe Storage Shelves**	Once a week	EVS	High Duster	Start at top and work down
Equipment and Miscellaneous				
**Removal of trash**	Once a shift	EVS	N/A	Place trash in proper container

NYPH CSPD Cleaning Standards and Responsibilities

**Removal of sharps**	Twice a week (Scheduled pick up)	Stericycle* **EVS to coordinate with Stericycle*	N/A	Container should be secured in a caddy
**Wash Soiled utility and transport carts**	Once a day	CSPD	Put through cart washer	
**Descale washer, cart washer and instrument washers**	Once a week	CSPD	Descaling Product from manufacturer	
**Sanitize and detail-clean dirty Case Cart Elevator (site specific)**	Once a day	EVS	Bleach Wipes	Ceiling, walls, floor, elevator tracks, vents
**Wipe all mechanical washers, handles, doors and conveyers**	Once a day	CSPD	Hospital approved disinfectant	

Surface/Item	Frequency	Dept. Responsible	Recommended Product	Details
**Assembly (Prep and Pack)** **Includes Scope Reprocessing Room *(site specific)***				
Floors, Sinks				
**Sweep floors and baseboards**	Once a day	EVS	Microfiber mop	
**Wet mop floors**	Once a day	EVS	Microfiber mop and Hospital approved disinfectant	
**Auto-Scrub Floors**	Once a month	EVS	Hospital approved disinfectant	Move all furniture from side to side. Once finished, Power Washer is stored Properly
**Wash Anti-fatigue mats**	Once a week	CSPD	Put through cart washer	
**Disinfect all sinks, faucets and backsplashes**	Once a day	EVS	Hospital approved disinfectant	
**Wipe Soap and Purell Dispensers**	Once a day	EVS	Hospital approved disinfectant	Fill as needed
Ceiling, Walls, Doors				
**Spot clean walls**	Once a week	EVS	Hospital approved disinfectant	
**Wash doors, door panel, automatic door panel, door handles, and glass**	Once a day	EVS	Hospital approved disinfectant and Glass Cleaner	Glass to be cleaned with glass cleaner, door with Hospital approved disinfectant.
**Wipe door bells**	Once a day	EVS	PDI Sani Wipe (Purple top)	
**Clean and/or Replace Ceiling Tiles**	Once a month	EVS or Facilities (see detail)	Hospital approved disinfectant	EVS to clean; Facilities to replace tiles when wet within 72 hours. Use Hepa Cart
**Dust external Light Fixtures and/or covers**	Once a week	EVS	High Duster	
**Wipe Air vents (internal)**	As needed	EVS / Facilities		EVS to notify Facilities with Work Order
**Dust Air Ducts, Vents (external covers)**	Once a month (more often if visibly soiled)	EVS	High Duster	
Shelving, Workstations				
**Wipe Cabinets (exterior)**	Once a day	EVS	Hospital approved disinfectant	
**Wipe Workstations, Shelves**	Once a day	EVS	Hospital approved disinfectant	Includes Ledges, shelving
**Wipe workstation bins**	Once a week	CSPD	PDI Sani Wipe (Purple top)	Inside and outside

NYPH CSPD Cleaning Standards and Responsibilities

**Dust Workstation lamps**	Once a day	EVS	Microfiber	
**Dust compressed air hoses**	Once a week	EVS	Duster	
**Wipe compressed air hose nozzles**	Once a day	CSPD	PDI Sani Wipe (Purple top)	
**Wipe telephones**	Once a day	EVS	PDI Sani Wipe (Purple top)	On workstations and on walls
**Dust Storage Shelves**	Once a week	EVS	High Duster, Microfiber	Start at top and work down
**Wipe pass-through windows, shelves and doors**	Once a day	EVS	Hospital approved disinfectant	
Equipment and Miscellaneous				
**Removal of trash**	Once a shift	EVS	N/A	Place trash in proper container
**Removal of sharps**	Twice a week (Scheduled pick up)	Stericycle* **EVS to coordinate with Stericycle*		Container should be secured in a caddy
**Wash Transport carts**	Once a week	CSPD	Put through cart washer	
**Wipe printers**	Once a week	EVS	PDI Sani Wipe (Purple top)	
**Wipe all mechanical washers, handles, doors and conveyers**	Once a day	CSPD	PDI Sani Wipe (Purple top)	

Surface/Item	Frequency	Dept. Responsible	Recommended Product	Details
**Sterilization and Ste ile Storage / Supply Room** (site specific)				
Floors, Sinks				
**Sweep floors and baseboards**	Once a day	EVS	Microfiber mop	
**Wet mop floors**	Once a day	EVS	Microfiber mop and Hospital approved disinfectant	
**Auto-Scrub floors**	Once a month	EVS	Hospital approved disinfectant	Move all furniture from side to side. Once finished, Power Washer is stored Properly
**Clean Anti-fatigue mats**	Once a week	CSPD	Put through cart washer	
**Disinfect all sinks, faucets and backsplashes**	Once a day	EVS	Hospital approved disinfectant	
**Wipe Soap and Purell Dispensers**	Once a day	EVS	Hospital approved disinfectant	Fill as needed
Ceiling, Walls, Doors				
**Spot clean walls**	Once a week	EVS	Hospital approved disinfectant	
**Wash doors, door panel, automatic door panel, door handles, and glass**	Once a day	EVS	Hospital approved disinfectant and Glass Cleaner	Glass to be cleaned with glass cleaner, door with Hospital approved disinfectant.
**Clean and/or Replace Ceiling Tiles**	Once a month	EVS or Facilities (see detail)	Hospital approved disinfectant	EVS to clean; Facilities to replace tiles when wet within 72 hours. Use Hepa Cart
**Dust external Light Fixtures and/or covers**	Once a week	EVS	High Duster	

NYPH CSPD Cleaning Standards and Responsibilities

**Wipe Air vents (internal)**	As needed	EVS / Facilities		EVS to notify Facilities with Work Order
**Dust Air Ducts, Vents (external covers)**	Once a month (more often if visibly soiled)	EVS	High Duster	
Shelving, Workstations				
**Wipe Cabinets (exterior)**	Once a day	EVS	Hospital approved disinfectant	
**Wipe Storage Shelves**	Once a day	EVS	Hospital approved disinfectant	
**Dust instrument storage shelves**	Once a week	CSPD	High Duster, Microfiber	Start at top and work down
**Wipe sterile instrument (peel pack) bins and drawers**	Once a week	CSPD	PDI Sani Wipe (Purple top)	
**Dust Workstation lamps**	Once a day	EVS	High Duster, Microfiber	
**Wipe printers**	Once a week	EVS	PDI Sani Wipe (Purple top)	
**Wipe telephones**	Once a day	EVS	PDI Sani Wipe (Purple top)	
Equipment and Miscellaneous				
**Removal of trash**	Once a shift	EVS	N/A	Place trash in proper container
**Wash Transport carts**	Once a week	CSPD	Put through cart washer	
**Sanitize and detail clean Case Cart Elevator (site specific)**	Twice a week	EVS	Hospital approved disinfectant	Ceiling, walls, floor, elevator tracks, vents
**Mop behind the sterilizers**	Once a week	EVS	Hospital approved disinfectant	
**Wipe and mop under Sterrad Sterilizers**	Once a month	*EVS Supervisor and *CSPD Supervisor	Hospital approved disinfectant	*Coordinated between supervisors
